# Diosgenin protects against cationic bovine serum albumin-induced membranous glomerulonephritis by attenuating oxidative stress and renal inflammation via the NF-κB pathway

**DOI:** 10.1080/13880209.2024.2330602

**Published:** 2024-03-22

**Authors:** Shiyan Jia, Ruihua Si, Guangzhen Liu, Qiming Zhong

**Affiliations:** a Department of Anesthesiology, Anesthesia and Trauma Research Unit, Hebei Cangzhou Hospital of Integrated Traditional Chinese Medicine and Western Medicine, Cangzhou, China; b College of Basic Medical Sciences, Shanxi University of Chinese Medicine, Jinzhong, China; c Department of Nephrology, Shanxi Province Hospital of Traditional Chinese Medicine, Taiyuan, China

**Keywords:** Chronic kidney disease, podocyte, traditional Chinese medicine, Nrf2/Keap1 signalling pathway

## Abstract

**Context:**

Membranous glomerulonephritis (MGN) is a leading cause of nephrotic syndrome in adults. Diosgenin (DG) has been reported to exert antioxidative and anti-inflammatory effects.

**Objective:**

To investigate the renoprotective activity of DG in a cationic bovine serum albumin-induced rat model of MGN.

**Materials and methods:**

Fourty male Sprague-Dawley rats were randomized into four groups. The MGN model was established and treated with a DG dose (10 mg/kg) and a positive control (TPCA1, 10 mg/kg), while normal control and MGN groups received distilled water by gavage for four consecutive weeks. At the end of the experiment, 24 h urinary protein, biochemical indices, oxidation and antioxidant levels, inflammatory parameters, histopathological examination, immunohistochemistry and immunoblotting were evaluated.

**Results:**

DG significantly ameliorated kidney dysfunction by decreasing urinary protein (0.56-fold), serum creatinine (SCr) (0.78-fold), BUN (0.71-fold), TC (0.66-fold) and TG (0.73-fold) levels, and increasing ALB (1.44-fold). DG also reduced MDA (0.82-fold) and NO (0.83-fold) levels while increasing the activity of SOD (1.56-fold), CAT (1.25-fold), glutathione peroxidase (GPx) (1.55-fold) and GSH (1.81-fold). Furthermore, DG reduced Keap1 (0.76-fold) expression, Nrf2 nuclear translocation (0.79-fold), and induced NQO1 (1.25-fold) and HO-1 (1.46-fold) expression. Additionally, DG decreased IL-2 (0.55-fold), TNF-α (0.80-fold) and IL-6 (0.75-fold) levels, and reduced protein expression of NF-κB p65 (0.80-fold), IKKβ (0.93-fold), p-IKKβ (0.89-fold), ICAM-1 (0.88-fold), VCAM-1 (0.91-fold), MCP-1 (0.88-fold) and E-selectin (0.87-fold), and also inhibited the nuclear translocation of NF-κB p65 (0.64-fold).

**Discussion and conclusions:**

The results suggest a potential therapeutic benefit of DG against MGN due to the inhibition of the NF-κB pathway, supporting the need for further clinical trials.

## Introduction

Membranous glomerulonephritis (MGN) is a primary cause of pathological nephrotic syndrome in adults, with up to 40% of patients eventually progressing towards end-stage renal failure within 10–15 years (Alsharhan and Beck 2021; Wu L et al. 2021). MGN is characterized by the progressive loss of renal function, podocyte injury, persistent inflammation, oxidative stress, and glomerular and tubulointerstitial fibrosis (Liu M et al. 2015). A number of prior studies have demonstrated that animal models of MGN present with glomerular damage, elevated oxidative stress due to enhanced generation of reactive oxygen species (ROS), diminished synthesis of antioxidant compounds, and the stimulation of pro-inflammatory mediators (Li et al. 2014; Wu J et al. 2016). Further, excessive ROS production by inflammatory nephritic cells can stimulate apoptosis, exacerbate inflammation and accelerate nephritis progression. Ample evidence indicates that oxidative stress and inflammation interplay significantly contributes to kidney injury (Rojas-Rivera et al. 2012; Yang ZJ et al. 2019). Despite several drugs having been developed to treat MGN in recent years, their limited efficacy and severe side effects have been frequently reported (du Buf-Vereijken et al. 2005; Fiorentino et al. 2016; Luan et al. 2020). Consequently, there is an urgent need to identify novel therapeutic approaches that offer improved efficacy and safety profiles.

Currently, compounds isolated from natural products or traditional Chinese medicines have shown beneficial effects in treating MGN (Hua et al. 2023). Astragaloside IV can alleviate podocyte injury by inhibiting renin–angiotensin system (RAS) signalling via the Wnt1/β-catenin pathway, thus presenting its potential role as a β-catenin inhibitor for MGN treatment (Wang YN et al. 2023). Similarly, salvianolic acid B activates renal autophagy by targeting the phosphoinositol-3 kinase (PI3K)/protein kinase B (AKT) pathway through microRNA-145-5p, consequently reducing inflammation and cell proliferation (Chen J et al. 2022). Additionally, salvianolate has been found to protect against MGN by inhibiting hypercoagulable states and promoting the expression of Wilms’ tumour protein 1 (WT-1), podocalyxin (PCX) and vascular endothelial growth factor (VEGF) in renal tissue, which ultimately aids in the repair of podocyte injury (Chen W et al. 2023). Diosgenin (DG) is a prominent steroid sapogenin extracted from various medicinal plants, including *Solanum* (Solanaceae), *Dioscorea* (Dioscoreaceae) and *Costus* (Zingiberaceae) species, and has been reported to offer numerous biological benefits (Guo et al. 2019). DG has been suggested to possess therapeutic effects in diverse disorders, such as those related to inflammatory and oxidative stress responses, diabetes, cardiovascular diseases and cancer (Wankhede et al. 2016; Song et al. 2017; Cai et al. 2020). Owing to its structure, DG functions in multiple ways, either as a precursor to steroid hormones such as progesterone and testosterone, both with anti-inflammatory properties, or as a genetic regulator, upregulating anti-apoptotic and antioxidant genes (Sato et al. 2014; Sirotkin et al. 2019). Previous research from our laboratory has demonstrated that DG possesses potent antioxidant properties capable of effectively protecting renal glomerular epithelial cells, while other studies have shown that DG suppresses lipopolysaccharide-induced inflammatory responses in murine macrophages through the inhibition of inflammatory mediators (Wang R et al. 2015; Yan and Liu 2017).

However, the protective effects of DG in MGN have not been thoroughly investigated. Consequently, this study evaluates the renoprotective effects of DG in a cationic bovine serum albumin (C-BSA)-induced rat model of MGN and explores the potential underlying mechanisms with a focus on oxidative stress and inflammation.

## Materials and methods

### Chemicals

Diosgenin (purity >98%, cat. no. D1634), [(aminocarbony)amino]-5-(4-fluorophenyl)-3-thiophenecarboxamide (TPCA1, IKKβ inhibitor, purity >98%, cat. no. T1452) and C-BSA (purity >98%, cat. no. A1933) were purchased from Sigma-Aldrich (St. Louis, MO). Anti-rabbit antibodies against nephrin (cat. no. ab216341, RRID: AB_2864307), podocin (cat. no. ab181143, RRID: AB_2885014), glyceraldehyde 3-phosphate dehydrogenase (GAPDH, cat. no. ab181602, RRID: AB_2630358), heme oxygenase 1 (HO-1, cat. no. ab68477, RRID: AB_1267209), NAD(P)H:quinone oxidoreductase-1 (NQO-1, cat. no. ab80588, RRID: AB_2924407), histone H2B (cat. no. ab52599, RRID: AB_880435), intercellular adhesion molecule 1 (ICAM-1, cat. no. ab282575), vascular cell adhesion protein 1 (VCAM-1, cat. no. ab134047, RRID: AB_2895043) and monocyte chemoattractant protein 1 (MCP-1, cat. no. ab7202) were purchased from Abcam (Cambridge, MA). Anti-rabbit antibodies against nuclear factor erythroid 2-related factor 2 (Nrf2, cat. no. SAB4501984, RRID: AB_10747179) and Kelch-like ECH-associated protein 1 (Keap1, cat. no. SAB5701091) were purchased from Sigma-Aldrich (St. Louis, MO). Anti-rabbit antibodies against nuclear factor-κB p65 (NF-κB p65, cat. no. D14E12, RRID: AB_2799359), IκB kinase-β (IKKβ, cat. no. D30C6) and p-IKKβ (cat. no. 16A6) were purchased from Cell Signaling Technology (Danvers, MA). Anti-rabbit antibody against E-selectin was purchased from Absin Bioscience (cat. no. abs13634, Shanghai, China). All other chemicals and reagents used in the study were acquired from commercially available sources.

### Animals

Fourty male Sprague-Dawley (SD) rats (6–8 weeks, 200–220 g) were purchased from the National Institutes for Food and Drug Control (certification no. SCXK(Jing) 2017-0005, Beijing, China). The rats were housed under standard conditions with a 12 h light/dark cycle, controlled temperature (22 ± 2 °C) and relative humidity (45–55%). The experiment was conducted in the central laboratory of Shanxi Provincial Hospital of Traditional Chinese Medicine (Taiyuan, China). All animal studies were conducted following the National Institutes of Health Guide for Laboratory Animal Care and Use and approved by the Animal Ethics Committee of Shanxi Provincial Hospital of Traditional Chinese Medicine (approval number: SZYLY2021KY-0811).

### Experimental design

We established a rat MGN model according to previous studies (Wu J et al. 2016; Gai et al. 2018). After one week of adaptive feeding, 30 rats were administered C-BSA to establish the MGN rat model, following the modified Border method (Border et al. 1982). In the pre-immunization phase, 1 mg of C-BSA was dissolved in 0.5 mL of normal saline and emulsified with an equal amount of incomplete Freund’s adjuvant, forming a milky white suspension. Multiple subcutaneous injections were administered to the rats’ neck, groin and armpit regions, 0.1 mL/rat, once every other day, for a total of three injections. In the formal immunization phase, C-BSA was combined with an equal volume of phosphate buffer and injected into the rat tail vein, 16 mg/kg, once every other day, three times a week for 4 weeks. The MGN condition in rats was confirmed by determining 24 h proteinuria levels using the Bradford assay kit (Sigma-Aldrich, St. Louis, MO), and successfully induced rats proceeded to the main study.

All successfully established MGN rats were separated randomly into three groups, each of 10 rats. Another 10 rats were served as normal control (NC) group. Normal control group were treated with distilled water daily; MGN control group (MGN) were treated with distilled water daily; DG group (MGN + DG) were treated with 10 mg/kg DG daily; TPCA1 group (MGN + TPCA1) were treated with 10 mg/kg TPCA1 daily. DG was dissolved in distilled water, and TPCA1 was dissolved in dimethyl sulphoxide; both drugs were administered orally to MGN rats for 4 consecutive weeks.

### Urine and serum collection and renal biochemical analysis

After the 4-week treatment period, the animals were placed in individual metabolic cages for 24 h urine collection. Urine samples were then centrifuged at 3000 rpm for 10 min to remove the debris, and supernatants were stored at −80 °C for further analysis. Blood samples were collected from the abdominal aorta under anaesthesia. Serum was separated by centrifugation at 3000 rpm for 10 min and stored at −20 °C. Rats were then euthanized by administering an overdose of pentobarbital sodium (150 mg/kg) followed by cervical dislocation, and kidney tissues were collected. A part of the kidney tissue was fixed in 10% formalin for histological examination, and the remaining kidney tissue was snap-frozen in liquid nitrogen and stored at −80 °C for later analysis. Urine protein levels and serum indexes, including serum creatinine (SCr), triglyceride (TG), total cholesterol (TC), albumin (ALB), blood urea nitrogen (BUN), were measured by using an automatic biochemical analyser (Hitachi 7600, Tokyo, Japan).

### Histopathological analysis

The 10% formalin-fixed kidney tissues underwent dehydration, paraffin embedding and sectioning at 4 μm thickness. The sections were stained with haematoxylin and eosin (HE) for morphological evaluation. Morphological changes were observed under a light microscope (Olympus BX50, Tokyo, Japan).

### Transmission electron microscopy

Kidney tissues were fixed in 2.5% glutaraldehyde solution, rinsed in phosphate-buffered saline (PBS), fixed with 1% osmic acid, dehydrated through acetone, embedded, polymerized, sectioned and double stained with uranyl acetate and lead acid. The sections were then examined using a Hitachi HT7700 electron microscope (Hitachi, Tokyo, Japan) operated at 60 kV with an absolute magnification of ×5000.

### Oxidative and antioxidative status analysis

Renal cortices were homogenized and centrifuged, and the supernatant obtained was used for the determination of oxidative status, including glutathione peroxidase (GPx), glutathione (GSH), catalase (CAT), nitric oxide (NO), superoxide dismutase (SOD) and malondialdehyde (MDA) levels. MDA levels were measured by thiobarbituric acid reactive substances (TBARS) assay (R&D Systems, Minneapolis, MN), while SOD, NO, CAT, GSH and GSH-Px activities were assessed using commercial assay kits obtained from Nanjing Biotechnology Co. Ltd. (Nanjing, China).

### Renal inflammatory markers analysis

Inflammatory cytokines levels, including interleukin-2 (IL-2), interleukin-6 (IL-6) and tumour necrosis factor-α (TNF-α), were measured in renal homogenate using enzyme-linked immunosorbent assay (ELISA) kits (R&D Systems, Minneapolis, MN) according to the manufacturer’s instructions.

### Immunofluorescence staining

Tissue slices were permeabilized with 0.5% Triton X-100 for 30 min, followed by blocking with 1 × PBS containing 5% goat serum and 0.3% Triton X-100 for 1 h at room temperature. Subsequently, tissue slices were incubated with anti-NF-κB p65 rabbit antibody (1:500 dilution, cat. no. 8242, RRID: AB_10859369, Cell Signaling Technology, Danvers, MA) at 4 °C overnight. After washed with PBS, tissue slices were incubated in anti-rabbit IgG (H + L), F(ab′)_2_ Fragment (Alexa Fluor^®^ 594 conjugate) (1:500 dilution, cat. no. 8889, Cell Signaling Technology, Danvers, MA) in dark for 1 h at room temperature and counterstained with 4′,6-diamidino-2-phenylindole (DAPI, Sigma-Aldrich, St. Louis, MO). Stained slices were observed using a confocal system (IX81, Olympus, Shinjuku, Japan).

### Western blotting

Total protein was extracted with radioimmunoprecipitation assay (RIPA) buffer (cat. no. 89900, Thermo Fisher Scientific, Waltham, MA). Nuclear and cytosolic proteins were extracted using the NE-PER Nuclear and Cytoplasmic Extraction Reagents Kit (cat. no. 78835, Thermo Fisher Scientific, Waltham, MA) according to the protocol provided by the manufacturer. Protein samples (20 µg) were mixed with 5× loading buffer, denatured at 100 °C for 5 min, and electrophoresed on 10% sodium dodecyl sulphate polyacrylamide gel electrophoresis (SDS-PAGE) gels for 45 min. The proteins were then transferred to polyvinylidene fluoride (PVDF) membranes for 90 min and blocked with 5% bovine serum ALB for 60 min. Membranes were incubated with anti-rabbit primary antibodies (nephrin, podocin, GAPDH, HO-1, NQO-1, histone H2B, ICAM-1, VCAM-1, MCP-1, Nrf2, Keap1, NF-κB p65, IKKβ, p-IKKβ and E-selectin; all antibodies were diluted 1:2000 for 120 min at room temperature, washed and then incubated with HRP-linked goat anti-rabbit IgG (1:5000 dilution, cat. no. ab6721, Abcam, Cambridge, MA) for 30 min at room temperature. After the final wash, membranes were detected using an enhance chemiluminescence (ECL) kit (Millipore, Burlington, MA) under a multifunctional imaging system (Tanon 5200, Shanghai, China). Band grey values were analysed using Image J software (National Institutes of Health, Bethesda, MD), and protein expression ­levels were normalized to the housekeeping genes GAPDH or histone H2B.

### Statistical analysis

Statistical analysis was performed using GraphPad Prism 9.0 software (GraphPad Software, Inc., San Diego, CA). Data were expressed as mean ± standard deviation (SD). Statistical analysis was performed using one-way analysis of variance (ANOVA) followed by Tukey’s multiple comparison *post hoc* test. *p* < 0.05 was considered statistically significant.

## Results

### Effects of DG on body weight and kidney index in MGN rats

**Figure 1.**
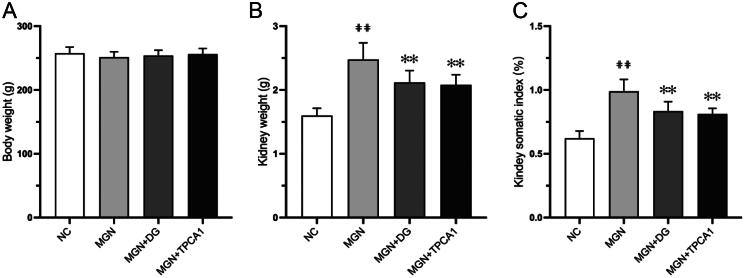
DG has no distinct toxic effects in MGN rats after 4 weeks treatment. (A) Body weight. (B) Kidney weight. (C) Kidney somatic index. ^##^*p* < 0.01 vs. normal control group. Data are expressed as the mean ± standard deviation (SD), *n* = 6. **p < 0.01 vs. MGN group. NC: normal control; MGN: membranous glomerulonephritis; DG: diosgenin; TPCA1: [(aminocarbony)amino]-5-(4-fluorophenyl)-3-thiophenecarboxamide. The somatic index is defined as follows: somatic index= kidney weight (g)/body weight (g).

We first evaluated the toxicity of DG on rats. As shown in Figure 1(A), there was no significant difference observed in the body weight of MGN rats compared with NC rats. Conversely, a significant increase in kidney weight (*p* < .01; Figure 1(B)) and somatic index (*p* < .01; Figure 1(C)) was observed in MGN rats compared with NC rats. Treatment of MGN rats with DG or TPCA1 significantly reduced the kidney weight and somatic index.

### DG improves renal function and biochemical parameters in MGN rats

**Figure 2.**
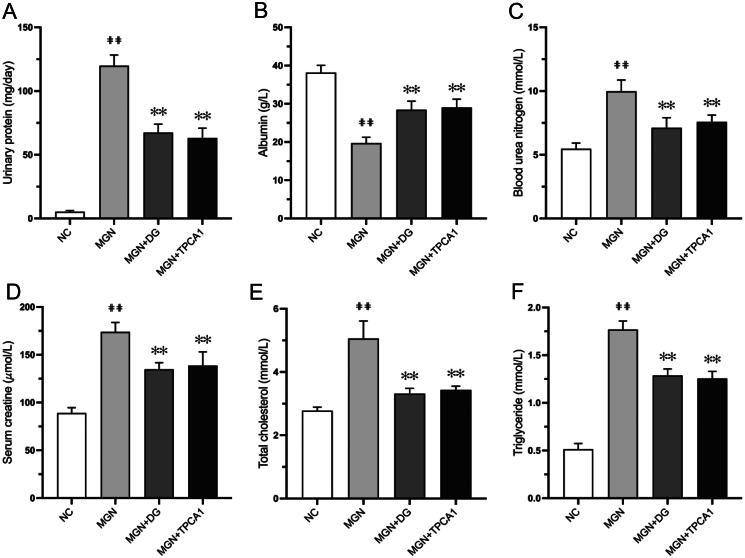
DG improves 24 h urinary protein and serum biochemical parameters in MGN rats. (A) Urinary protein. (B) Albumin (ALB). (C) Blood urea nitrogen (BUN). (D) Serum creatinine (SCr). (E) Total cholesterol (TC). (F) Triglyceride (TG). Data are expressed as the mean ± standard deviation (SD), *n* = 6. ^##^*p* < 0.01 vs. NC group. **p < 0.01 vs. MGN group. NC: normal control; MGN: membranous glomerulonephritis; DG: diosgenin; TPCA1: [(aminocarbony)amino]-5-(4-fluorophenyl)-3-thiophenecarboxamide.

The C-BSA-induced MGN rat model mirrors the clinical and pathological features of human MGN (Zhao et al. 2022). Consistent with the marked changes in proteinuria, MGN rats had significantly increased urinary protein level (*p* < 0.01; Figure 2(A)). Additionally, the level of ALB was significantly decreased (*p* < 0.01; Figure 2(B)) with increased levels of BUN, SCr, TC and TG (*p* < 0.01; Figure 2(C–F)) compared with NC rats. Treatment with either DG or TPCA1 significantly reduced the urinary protein, SCr, BUN, TC and TG levels and significantly increased ALB compared with untreated MGN rats.

### DG attenuates glomerular damage in MGN rats

**Figure 3.**
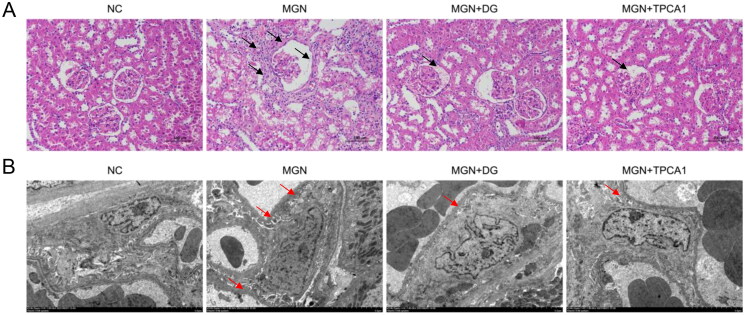
Effects of DG on histopathological changes in MGN rats. (A) Representative haematoxylin and eosin (H&E) staining of kidney samples (original magnification ×200; scale bar represents 100 μm). (B) Transmission electron micrographs of renal tissue (original magnification ×5000; scale bar represents 5 μm). Black arrows represent oedema and vacuolar degeneration of renal tubular epithelial cells and glomerular atrophy. Red arrows represent the presence of glomerular podocyte fusion and electron-dense deposits in the GBM and epithelia. NC: normal control; MGN: membranous glomerulonephritis; DG: diosgenin; TPCA1: [(aminocarbony)amino]-5-(4-fluorophenyl)-3-thiophenecarboxamide.

Renal morphology was examined by H&E staining and transmission electron microscopy (TEM) (Figure 3). H&E staining revealed that NC rats displayed normal renal tissue morphology, with intact basement membranes of glomerular capillaries and tubular epithelium, and normal-sized mesangium. In contrast, MGN rats exhibited thick glomerular basement membrane (GBM) and glomerular atrophy (Figure 3(A)). TEM revealed glomerular podocyte fusion, mesangial cell proliferation, increased matrix and electron-dense deposits in both the GBM and epithelia (Figure 3(B)). However, treatment with DG or TPCA1 suppressed the abnormal increase in inflammatory cell infiltration and glomerular expansion, and ameliorated the foot process fusion and GBM thickening.

### DG restores nephrin and podocin expression in MGN rats

**Figure 4.**
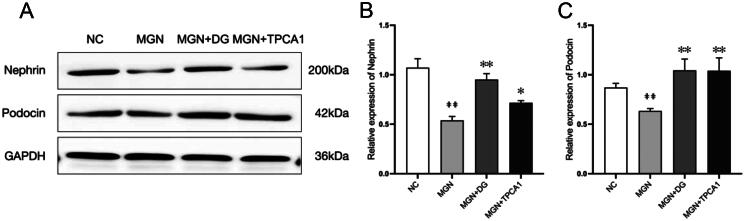
DG restores nephrin and podocin expression levels in MGN rats. (A) Protein levels of nephrin and podocin were evaluated by western blotting. (B, C) Ratio of nephrin and podocin. Data are expressed as the mean ± standard deviation (SD), *n* = 3. ^##^*p* < 0.01 vs. NC group. *p < 0.05 or **p < 0.01 vs. MGN group. NC: normal control; MGN: membranous glomerulonephritis; DG: diosgenin; TPCA1: [(aminocarbony)amino]-5-(4-fluorophenyl)-3-thiophenecarboxamide.

Nephrin and podocin are podocyte‐specific molecules that play a crucial role in maintaining the normal structure and function of the glomerular filtration membrane (Xie et al. 2020). The results demonstrated significant decreases in nephrin and podocin protein expression levels in MGN rat podocytes compared with NC rats (*p* < 0.01, Figure 4). Subsequently, protein levels of nephrin and podocin were significantly restored in MGN rats following DG or TPCA1 treatment.

### DG ameliorates oxidative stress in MGN rats

**Figure 5.**
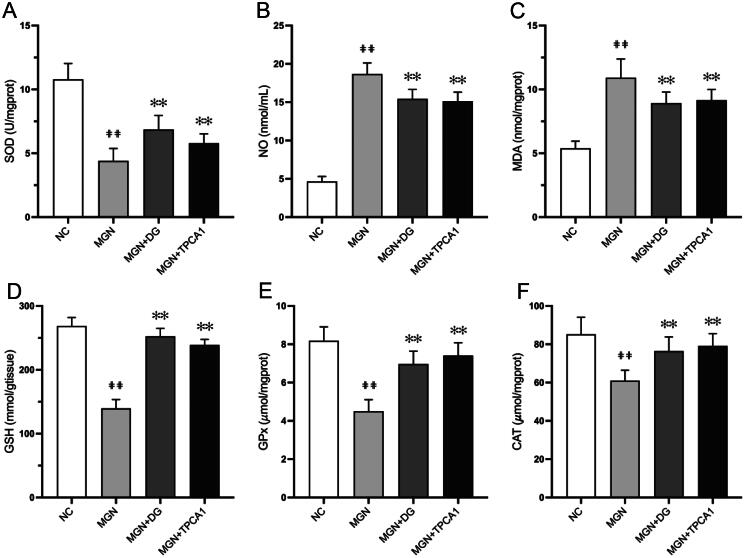
Effects of DG on renal oxidant and antioxidant levels in MGN rats. (A) SOD, (B) NO, (C) MDA, (D) GSH, (E) GPx, (F) CAT. Data are expressed as the mean ± standard deviation (SD), *n* = 3. ^##^*p* < 0.01 vs. NC group. **p < 0.01 vs. MGN group. SOD: superoxide dismutase; NO: nitric oxide; MDA: malondialdehyde; GPx: glutathione peroxidase; CAT: catalase; NC: normal control; MGN: membranous glomerulonephritis; DG: diosgenin; TPCA1: [(aminocarbony)amino]-5-(4-fluorophenyl)-3-­thiophenecarboxamide.

To explore the potential mechanism underlying the protective effect of DG on the kidneys challenged by C-BSA, renal oxidant and antioxidant levels were assessed. Renal lipid peroxidation products, NO and MDA levels were significantly elevated (*p* < 0.01; Figure 5(B,C)) in MGN rats compared with NC rats. Conversely, renal SOD, GSH, GPx and CAT activity levels were significantly reduced (*p* < 0.01; Figure 5(A,D–F)). Notably, DG- or TPCA1-treated MGN rats exhibited significantly decreased levels of MDA and NO associated with significantly increased activity levels of SOD, CAT, GPx and GSH when compared with untreated MGN rats.

### DG modulated the Nrf2/Keap1 signalling pathway in MGN rats

**Figure 6.**
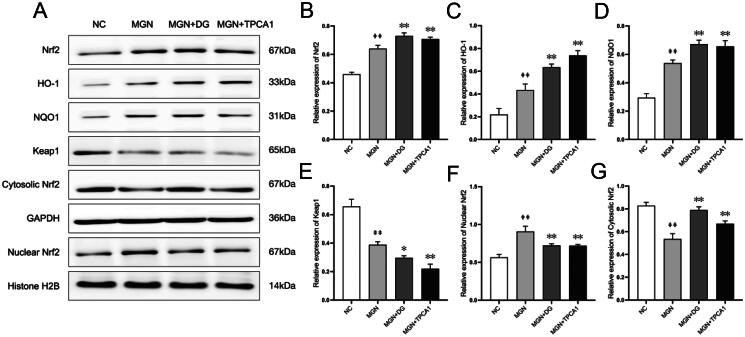
DG activates the Nrf2 signalling pathway in MGN rats. (A) Protein levels of Nrf2, HO-1, NQO1, Keap1, nuclear Nrf2 and cytosolic Nrf2 were evaluated by western blotting. (B–G) Ratio of Nrf2, HO-1, NQO1, Keap1, nuclear Nrf2 and cytosolic Nrf2. Data are expressed as the mean ± standard deviation (SD), *n* = 3. ^##^*p* < 0.01 vs. NC group. *p < 0.05 or **p < 0.01 vs. MGN group. NC: normal control; MGN: membranous glomerulonephritis; DG: diosgenin; TPCA1: [(aminocarbony)amino]-5-(4-fluorophenyl)-3-thiophenecarboxamide.

Considering the observed antioxidative effects of DG, we aimed to investigate its influence on the Nrf2/Keap1 signalling pathway (Figure 6). Western blot analysis showed that the expression levels of Nrf2 and its downstream antioxidative enzymes, NQO1 and HO-1, were significantly downregulated in MGN rats compared with the NC rats (*p* < 0.05, *p* < 0.01; Figure 6(B–D)). Conversely, Keap1, a negative regulator of Nrf2, was upregulated in MGN rats (*p* < 0.01; Figure 6(E)). Furthermore, DG treatment notably increased Nrf2, NQO1 and HO-1 expression levels while decreasing Keap1 expression in MGN rats. The present study results also confirmed that a significantly increased nuclear Nrf2 (*p* < 0.01; Figure 6(F)) with decreased cytosolic Nrf2 (*p* < 0.01; Figure 6(G)) protein expression levels was observed in MGN rats. However, DG or TPCA1 treatment resulted in a further downregulation of nuclear Nrf2 and upregulation of cytosolic Nrf2 expression. These results suggest that the antioxidative effects of DG in MGN rats are potentially mediated through the modulation of the Nrf2/Keap1 signalling pathway.

### DG exhibits anti-inflammatory effects via inhibition of the NF-κB signalling pathway in MGN rats

**Figure 7.**
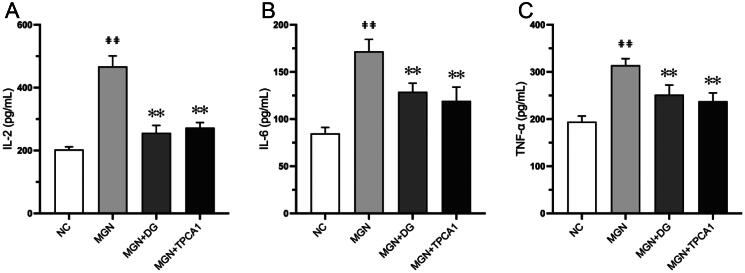
DG inhibits inflammatory cytokines secretion in MGN rats. (A) IL-2, (B) IL-6, (C) TNF-α. Data are expressed as the mean ± standard deviation (SD), *n* = 6. ^##^*p* < 0.01 vs. NC group. **p < 0.01 vs. MGN group. IL-2: interleukin-2; IL-6: interleukin-6; TNF-α: tumor necrosis factor-α; NC: normal control; MGN: membranous glomerulonephritis; DG: diosgenin; TPCA1: [(aminocarbony)amino]-5-(4-fluorophenyl)-3-thiophenecarboxamide.

**Figure 8.**
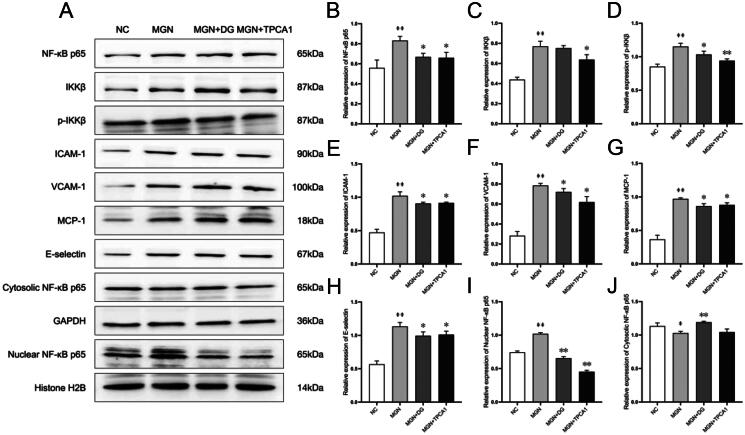
DG inactivates of the NF-κB signalling pathway in MGN rats. (A) Protein levels of NF-κB p65, IKKβ, p-IKKβ, ICAM-1, VCAM-1, MCP-1, E-selectin, nuclear NF-κB p65 and cytosolic NF-κB p65 were evaluated by western blotting. (B–J) Ratio of NF-κB p65, IKKβ, p-IKKβ, ICAM-1, VCAM-1, MCP-1, E-selectin, nuclear NF-κB p65 and cytosolic NF-κB p65. Data are expressed as the mean ± standard deviation (SD), *n* = 3. ^#^*p* < 0.05 or ^##^*p* < 0.01 vs. NC group. *p < 0.05 or **p < 0.01 vs. MGN group. NC: normal control; MGN: membranous glomerulonephritis; DG: diosgenin; TPCA1: [(aminocarbony)amino]-5-(4-fluorophenyl)-3-thiophenecarboxamide.

**Figure 9.**
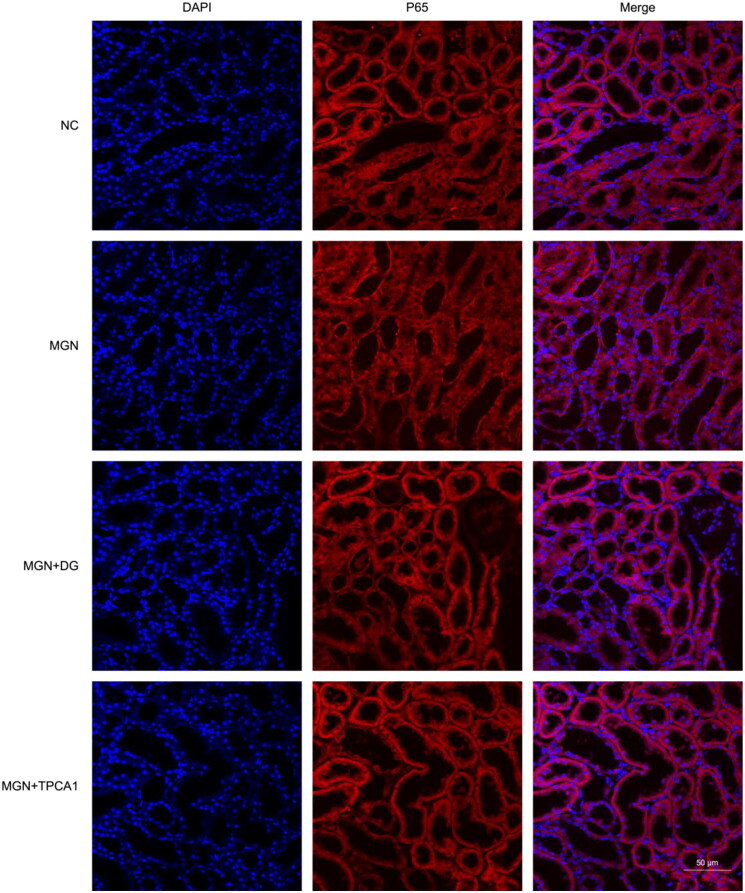
Effect of DG on NF-κB p65 nuclear translocation in MGN rats. NF-κB p65 expression was detected using immunofluorescence staining. NC: normal control; MGN: membranous glomerulonephritis; DG: diosgenin; TPCA1: [(aminocarbony)amino]-5-(4-fluorophenyl)-3-thiophenecarboxamide. Scale bar: 50 μm.

The effect of DG treatment on inflammatory cytokine levels is shown in Figure 7. MGN rats exhibited significant increase in IL-2, TNF-α and IL-6 levels compared with NC rats (*p* < .01; Figure 7(A–C)). Oral administration of DG markedly reduced these elevated cytokine levels, with similar regulatory effects observed for TPCA1. To explore the anti-inflammatory properties of DG, we assessed its influence on the NF-κB signalling pathway. Western blot analysis revealed significantly upregulated expression levels of NF-κB p65, IKKβ, p-IKKβ, ICAM-1, VCAM-1, MCP-1 and E-selectin in MGN rats compared with the NC rats (*p* < 0.01; Figure 8(B–H)). DG treatment suppressed the expression levels of these pro-inflammatory markers in MGN rats. Additionally, western blot analysis results also confirmed that increased nuclear (*p* < 0.01; Figure 8(I)) and decreased cytosolic (*p* < 0.05; Figure 8(J)) NF-κB p65 protein levels of MGN rats compared with NC rats. However, MGN rats treated with either DG or TPCA1 displayed significantly increased cytosolic and decreased nuclear NF-κB p65 protein levels when compared with untreated MGN rats. The immunofluorescence staining results confirmed the above results of NF-κB p65 nuclear translocation (Figure 9). These results confirm the anti-inflammatory effects of DG in MGN rats, which are mediated through the inhibition of the NF-κB signalling pathway.

## Discussion

In this study, we demonstrated the renoprotective effects of DG in a C-BSA-induced rat model of MGN. DG treatment significantly ameliorated kidney dysfunction, improved glomerular morphology and restored the expression of key proteins involved in the glomerular filtration barrier, nephrin and podocin. Furthermore, we found that the renoprotective effects of DG were partly attributed to its ability to reduce oxidative stress and inflammation by modulating the Nrf2/Keap1 and NF-κB signalling pathways, respectively.

In the present study, no significant change in body weight was observed across treatment groups, suggesting minimal systemic toxicity. Although kidney weight and somatic index significantly increased in MGN rats compared with NC rats, this could be attributed to the inflammatory effects observed in the study. These results align with findings from previous studies (Sutariya et al. 2017). Conversely, treatment with DG or TPCA1 significantly restored the kidney weight and somatic index when compared with untreated MGN rats.

Proteinuria is usually considered to be representative of an injured filtration membrane and decreased renal function (Wang Y et al. 2019). Therefore, the proteinuria is usually considered as a sensitive indicator of MGN. Moreover, SCr, TG, TC, ALB and BUN can be used as evaluation parameters for the confirmation of renal functional and structural integrity (Liu L et al. 2018; Tong et al. 2019). MGN rats exhibited typical nephrotic syndrome manifestations, including massive proteinuria and hypoalbuminemia. After treatment with DG for 4 weeks, we observed a significant reduction in proteinuria and an increase in serum ALB levels, indicating improved renal function. Histopathological examination further confirmed these improvements, revealing alleviated glomerular damage in DG-treated MGN rats.

Glomerular podocytes play a critical role in kidney structure and urinary filtration (Qi et al. 2016). Podocyte foot processes surround the GBM, forming slit diaphragms between adjacent foot processes, and slit diaphragm dysfunction results in proteinuria (Nanri et al. 2009). As key functional molecules in the podocyte slit diaphragm, nephrin is located at the outer leaflet of plasma membranes of podocyte slit diaphragm, while podocin interacts with nephrin (Tryggvason et al. 2006). Previous studies have shown that lower nephrin and podocin expression in MGN rats compared with NCs (Lu et al. 2014). Histopathological assessments in our study revealed that mild foot process swelling and segmental fusion, with partial slit membrane reduction or loss in MGN rats. Nephrin and podocin protein expression levels were diminished in MGN rats compared with NCs and positively correlated with increased urinary protein excretion. Furthermore, DG treatment not only mitigated foot process effacement and GBM thickening but also recovered nephrin and podocin expression, reducing proteinuria.

It is well known that chronic inflammation and oxidative stress are well-established major mechanisms contributing to renal injury (Yang J et al. 2013). Evidence suggests that oxidative factor production is physiologically relevant as an essential step in inflammation (McNulty et al. 2011). In nephritis, overproduction of ROS and reactive nitrogen species by inflammatory cells can intensify inflammation, resulting in tissue damage and nephritis progression (Shah et al. 2007). Conversely, antioxidant enzymes, including SOD, GSH-Px and CAT, scavenge free radicals and prevent oxidative damage (Jiang et al. 2018). Our results showed that DG treatment markedly ameliorated oxidative stress in MGN rats, evidenced by decreased MDA and NO contents and enhanced activities of antioxidant enzymes, such as SOD, CAT, GSH and GPx.

Nevertheless, the mechanisms underlying the antioxidant activity of DG remain to be elucidated. There is considerable evidence that Nrf2 is a crucial transcription factor regulating the antioxidant response. Upon oxidative stress, Nrf2 dissociates from Keap1 and translocates to the nucleus to bind to antioxidant response elements (ARE), which is an enhancer element initiating the transcription of phase-II enzymes and antioxidant enzymes, such as NQO1, HO-1 and GPx (Wang G et al. 2014; Shi and Fu 2019). In this study, we found that DG treatment increased Nrf2 expression and its downstream antioxidative enzymes NQO1 and HO-1 while inhibiting Keap1 expression in MGN rats. These findings are supported by previous studies demonstrating that DG treatment induces the expression of NQO1 and HO-1 through activation of the Nrf2/Keap1 pathway (Wang R et al. 2015; Bloomfield et al. 2017). Additionally, our study further revealed upregulation of nuclear Nrf2 protein expression compared with cytosolic Nrf2 expression. Therefore, the antioxidant activity of DG may involve Nrf2 signalling pathway activation through Keap1 downregulation, Nrf2 nuclear translocation, and subsequently, activation of Nrf2-regulated genes such as NQO1 and HO-1.

The Nrf2 pathway was previously reported to modulate pro-inflammatory cytokine and chemokine overproduction, inhibiting NF-κB activation. ROS accumulation promotes NF-κB p65 nuclear translocation, activating the NF-κB signalling pathway and resulting in pro-inflammatory gene transcription (Han et al. 2014; Chu et al. 2016). Upon stimulation, IκBα is phosphorylated by IKKβ, releasing NF-κB, which translocates to the nucleus and activates various inflammatory cytokines, including IL-2, IL-6 and TNF-α (Wang Z et al. 2018). NF-κB also mediates the expression of pro-inflammatory cell adhesion molecules. Our study demonstrated that DG treatment significantly suppressed the expression levels of pro-inflammatory markers, such as ICAM-1, VCAM-1, MCP-1 and E-selectin of MGN rats. These markers are known to play critical roles in promoting leukocyte adhesion, infiltration and activation, thereby contributing to inflammatory injury in MGN (Vulesevic et al. 2016; Yang TL et al. 2018). Accumulating evidence has indicated that the activation of NF-κB signalling pathway plays a critical role in various kidney diseases, including MGN (Wang YN et al. 2022). Animal models of MGN have shown promising results in terms of reducing renal damage and dyslipidaemia by targeting this pathway (Miao et al. 2022). One example is the use of total coumarins from *Hydrangea paniculata* Siebold (Hydrangeaceae), which has been found to limit IL-10 production by inhibiting both the PI3K/AKT and NF-κB signalling pathways, resulting in alleviated renal damage and dyslipidaemia (Wang W et al. 2022). Another study has shown that treatment with Sanqi oral solution can reduce proteinuria levels, improve renal damage and restore podocyte injuries, primarily through the suppression of the NF-κB signalling pathway (Tian et al. 2019). In addition, Liu B et al. (2019) also demonstrated that Zhen Wu Tang can reduce urine protein levels and alleviate kidney damage by downregulating the expression levels of NLRP3, caspase-1 and IL-1β, which interestingly is accompanied by the decrease in NF-κB pathway activation. Moreover, the current study found that DG treatment inhibited the NF-κB signalling pathway, with DG-treated MGN rats displaying decreased NF-κB p65, IKKβ and p-IKKβ expression levels and reduced NF-κB p65 nuclear translocation, indicating that the anti-inflammatory effects of DG are mediated through the suppression of the NF-κB signalling pathway.

Our findings suggest that DG treatment protects against MGN through its antioxidative and anti-inflammatory properties, attributable to Nrf2/Keap1 pathway activation and NF-κB pathway downregulation. Furthermore, the restoration of nephrin and podocin expression by DG might contribute to stabilizing the glomerular filtration barrier and ameliorating proteinuria in MGN rats. Collectively, these results provide substantial evidence supporting the renoprotective effects of DG in MGN and its potential as a novel therapeutic agent for MGN.

## Conclusions

Our study demonstrated that the therapeutic effects of DG in rats on C-BSA induced MGN. The therapeutic effects are associated with DG not only ameliorated oxidative stress via upregulated Nrf2/HO-1 expression, but also effectively inhibited inflammatory responses via inhibition of the NF-κB signalling pathway. These findings suggest that DG could be a promising therapeutic candidate for treating MGN. However, this non-clinical research further warrants clinical studies to apply these results in humans.

## Data Availability

The datasets used and/or analysed during the current study are available from the corresponding author on reasonable request.
